# Sexual Dimorphism in the Selenocysteine Lyase Knockout Mouse

**DOI:** 10.3390/nu10020159

**Published:** 2018-01-31

**Authors:** Ashley N. Ogawa-Wong, Ann C. Hashimoto, Herena Ha, Matthew W. Pitts, Lucia A. Seale, Marla J. Berry

**Affiliations:** Department of Cell and Molecular Biology, John A. Burns School of Medicine, University of Hawaii, 651 Ilalo St., Honolulu, HI 96813, USA; anogawa@hawaii.edu (A.N.O.-W.); ahashimo@hawaii.edu (A.C.H.); hyh231@hawaii.edu (H.H.); mwpitts@hawaii.edu (M.W.P.); mberry@hawaii.edu (M.J.B.)

**Keywords:** Scly, selenium, selenoproteins

## Abstract

Selenium (Se) is an essential micronutrient known for its antioxidant properties and health benefits, attributed to its presence in selenoproteins as the amino acid, selenocysteine. Selenocysteine lyase (Scly) catalyzes hydrolysis of selenocysteine to selenide and alanine, facilitating re-utilization of Se for de novo selenoprotein synthesis. Previously, it was reported that male Scly^−/−^ mice develop increased body weight and body fat composition, and altered lipid and carbohydrate metabolism, compared to wild type mice. Strikingly, females appeared to present with a less severe phenotype, suggesting the relationship between Scly and energy metabolism may be regulated in a sex-specific manner. Here, we report that while body weight and body fat gain occur in both male and female Scly^−/−^ mice, strikingly, males are susceptible to developing glucose intolerance, whereas female Scly^−/−^ mice are protected. Because Se is critical for male reproduction, we hypothesized that castration would attenuate the metabolic dysfunction observed in male Scly^−/−^ mice by eliminating sequestration of Se in testes. We report that fasting serum insulin levels were significantly reduced in castrated males compared to controls, but islet area was unchanged between groups. Finally, both male and female Scly^−/−^ mice exhibit reduced hypothalamic expression of selenoproteins S, M, and glutathione peroxidase 1.

## 1. Introduction

Selenocysteine lyase (Scly) decomposes the 21st amino acid, selenocysteine, producing selenide and alanine. Scly deletion in mice (Scly^−/−^) leads to obesity, hyperinsulinemia, and reduced glucose tolerance on a Se adequate diet [1]. When challenged with a selenium (Se) deficient diet, Scly^−/−^ mice exhibited a more severe phenotype. Strikingly, the metabolic phenotype is more prominent in male mice.

Sex differences in Se function are well documented, but not well understood. In particular, Se appears to be critical to the male reproductive system. Although Se can be found in many tissues, a significant portion of dietary Se is routed to the testes, and under conditions of Se limitation, Se uptake is prioritized to the brain and testes [2]. Targeted genetic deletion of the Se transport protein, selenoprotein P (SELENOP) in mice results in male infertility, but female mice remain fertile [3]. Another feature of this sexual dimorphism is development of neurological dysfunction in the aforementioned males, which is more pronounced than in their female counterparts. Because of the Se salvaging capability of Scly and the presence of multiple Sec residues in SELENOP, it has been suggested that their concerted action is necessary for Se transport and delivery to tissues [4]. To study the consequences of knocking out both Se transport and salvaging, and to elucidate whether the two proteins appear to work in concert, we previously generated and characterized Scly^−/−^SELENOP^−/−^ mice. Se supplementation was required to produce viable double knockout litters. Even with Se supplementation, male Scly^−/−^SELENOP^−/−^ mice exhibit reduced survival and develop audiogenic seizures, producing a far worse phenotype than observed with the deletion of either single gene [5]. Strikingly, castration rescues Scly^−/−^SELENOP^−/−^ mice, suggesting that the testes competes for Se at the expense of neurological function [6].

Type 2 diabetes (T2D) is characterized by insulin resistance, and can lead to cardiovascular disease and other negative health consequences. Although the association between increased incidence of T2D and elevated Se levels in humans is controversial, it is nevertheless interesting that this association appears to be restricted to men [7]. We previously reported that female Scly^−/−^ mice have a less pronounced metabolic phenotype than their male counterparts [1], suggesting that sex differences in the regulation of Se metabolism may have direct consequences on energy metabolism. As our studies to date focused on male Scly^−/−^ mice, we undertook characterization of the metabolic phenotype of female Scly^−/−^ mice. We observed that although both male and female Scly^−/−^ mice develop an obese phenotype, female Scly^−/−^ mice were protected from obesity-induced glucose intolerance and hyperinsulinemia. We further hypothesized that the Se demands of the testes exert a detrimental effect on energy metabolism in male Scly^−/−^ mice. We therefore tested the effects of castration of male Scly^−/−^ mice on body weight, energy expenditure, and measures of insulin secretion and resistance. Castration did not affect energy expenditure in male Scly^−/−^ mice, but resulted in reduced decreased fasting serum insulin that could not be explained by differences in islet size.

Although Scly is implicated in lipid metabolism, white adipose tissue (WAT) does not appear to express Scly at high levels [1,8], inviting speculation that Scly acts through indirect mechanisms to regulate body fat. The hypothalamus is the master regulator of energy homeostasis, sensing nutrient availability and subsequently fine-tuning energy intake and expenditure through its downstream targets [9]. Impairment of selenoprotein synthesis through the deletion of Sec-tRNA^[Ser]Sec^ from the hypothalamus resulted in severe glucose intolerance and insulin resistance in mice [10], indicating that hypothalamic selenoprotein function is necessary to maintain energy metabolism. Nevertheless, little is known about the potential role of Scly in hypothalamic selenoprotein biosynthesis and function. Strikingly, hypothalamic selenoprotein expression was decreased in Scly^−/−^ mice. Taken together, our findings indicate a previously unestablished role for Scly in hypothalamic selenoprotein expression, with potential consequences for hypothalamic function.

## 2. Materials and Methods

### 2.1. Animals

Whole-body Scly^−/−^ mice were generated and backcrossed to wild-type (WT) C57BL/6N mice as previously described [11]. All mice were bred and housed in the animal facility at the University of Hawaii. Mice were weaned onto a special diet containing adequate (0.25 ppm) or low (0.08 ppm) levels of Se in the form of sodium selenite (Research Diets Inc., New Brunswick, NJ, USA) and maintained on a 12-h light/dark cycle. Unless otherwise noted, animals were euthanized via CO_2_ asphyxiation. All procedures were approved by the Institutional Animal Care and Use Committee of the University of Hawaii (09-871).

### 2.2. Castration

Five-week-old male mice were anesthetized with isofluorane (2 L/min) and a 1–2 cm ventral midline incision was made in the scrotum to expose the tunica. The tunica was pierced and the testes pushed out one at a time and excised, and all deferential vessels and ducts were replaced. Skin incisions were closed with 6.0 surgical silk. Animals were administered ibuprofen and allowed to recover for one week. Sham operations were also performed as controls.

### 2.3. Measurement of Metabolic Parameters

For the glucose tolerance test (GTT), mice were fasted overnight and injected intraperitoneally with glucose (1 g/kg of body weight). Tail vein blood was collected at 0, 30, 60, and 120 min post-injection and tested for glucose with a glucometer (LifeScan, Milpitas, CA, USA). For fasting insulin, tail blood was collected after a 4-hour fast. Serum insulin was measured using a mouse ultrasensitive insulin ELISA (ALPCO, Salem, NH, USA). To assess hepatic insulin signaling, mice were insulin challenged (10 mU/g of body weight) after a 4-hour fast. After CO_2_ asphyxiation, livers were collected, snap-frozen in liquid nitrogen, and stored at −80 °C until ready for processing.

### 2.4. Measurement of Energy Expenditure

Mice were habituated in the metabolic cages overnight, one week prior to metabolic testing, in order to reduce stress levels that can affect oxygen consumption. Food intake, activity, volume oxygen consumption (VO_2_), and volume carbon dioxide (VCO_2_) measurements were obtained over a 24 h period using the Panlab Oxylet*Pro*™ System (Harvard Apparatus, Barcelona, Spain). Data were analyzed using the Panlab METABOLISM software (Vídeňská, Prague, Czech Republic).

### 2.5. Immunohistochemistry and Islet Size Determination

Mice were anesthetized with Avertin and perfused with 4% paraformaldehyde. Pancreases were excised, embedded in paraffin, and sectioned at 5 μm thickness using a microtome (Leica; Wetzlar, Germany). Antigen unmasking was completed with boiling in 0.1 M citrate buffer, pH 6 (0.1 M anhydrous citric acid, 0.05% Tween-20) for 4 min. M.O.M. basic kit (Vector Labs, Burlingame, CA, USA) was used to stain for insulin according to manufacturer’s instructions. Endogenous peroxide activity was blocked using 3% H_2_O_2_ in water for 5 min. Vectastain Elite ABC kit (Vector Labs) and DAB substrate kit (Vector Labs) were used for detection according to manufacturer’s instructions. Hematoxylin was used as a counterstain to visualize nuclei. Anti-insulin was purchased from Millipore (Billerica, MA, USA). From each mouse, the area for 4–10 islets were calculated using ImageJ software (National Institutes of Health, Bethesda, MD, USA) [12].

### 2.6. Western Blot

Mouse whole brain was removed and incubated in ice-cold 0.32 M sucrose solution for 5 min before placing in a mouse brain matrix with 1-mm slice intervals, ventral side facing up. Using a razor blade, a 2-mm slice was cut starting at the optic chiasm. The section was placed on a filter paper and the hypothalamus was extracted using a scalpel under a dissecting scope. Tissues were re-suspended in Sigma CelLytic™ MT (St. Louis, MO, USA) with 1% Protease Inhibitor Cocktail III (Calbiochem; San Diego, CA, USA) and disrupted with a sonicator using three 10 s pulses over ice. Protein concentration was measured using the Bradford method with the Bio-Rad Protein Assay Reagent (Hercules, CA, USA). Twenty μg aliquots of total protein were separated on a 4–20% Tris-glycine polyacrylamide gel (Bio-Rad, Hercules, CA, USA), transferred to an Immobilon-FL membrane (Li-Cor Biosciences, Lincoln, NE, USA) and incubated in primary antibody overnight at 4 °C. Blots were washed three times for 5 min in 0.1% Tween-20 in phosphate-buffered saline and incubated in the appropriate IRDye secondary antibody (Li-Cor Biosciences, Lincoln, NE, USA) for 30 min. An Odyssey IR imaging system (Li-Cor Biosciences, Lincoln, NE, USA) was used for detection, and analysis was completed on the Image Studio v4.0 software (Li-Cor Biosciences, Lincoln, NE, USA). The antibodies used were anti-pACC1 Ser79, anti-ACC1, anti-pAkt Ser473, anti-Akt, anti-AMPKα, anti-pAMPKα Thr172 (Cell Signaling, Beverly, MA, USA), anti-GPX1 (R&D Systems, Inc., Minneapolis, MN, USA), anti-SELENOM (Sigma-Aldrich, St. Louis, MO, USA), anti-SELENOS (Sigma-Aldrich, St. Louis, MO, USA), anti-SEPHS2 (Rockland, Gilbertsville, PA, USA), and anti-TXNRD1 (NovusBio, Littleton, CO, USA).

### 2.7. Statistical Analyses

Unless otherwise noted, data were analyzed and graphed with GraphPad Prism 5 (GraphPad Software Inc., La Jolla, CA, USA) using the appropriate statistical tests. The statistical tests are referred to in figure legends.

## 3. Results

### 3.1. Female Scly^−/−^ Mice Have Increased Adiposity but Normal Fasting Insulin and Glucose Tolerance

**Figure 1:**
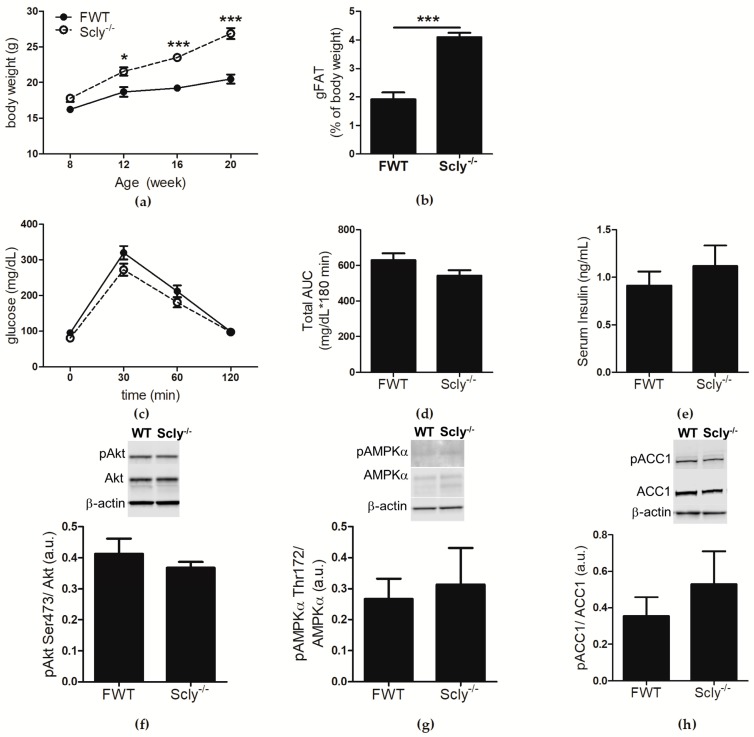
Metabolic characterization of female Scly^−/−^ mice on a Se deficient diet. For metabolic characterization, WT: *n* = 4, Scly^−/−^: *n* = 7. Unless otherwise indicated, mice were 22-weeks-old. (**a**) Body weight of female WT (FWT) and Scly^−/−^ mice at the indicated time points (two-way ANOVA: time *F*_3,27_ = 99.33, *p* < 0.0001, genotype *F*_1,27_ = 25.38, *p* = 0.0007, interaction *F*_3,27_ = 13.87, *p* < 0.0001); (**b**) gonadal WAT (gWAT) expressed as % body weight; (**c**) glucose tolerance test in 20-week-old mice; (**d**) area under the curve (AUC); (**e**) fasting serum insulin. Twenty-two-week old mice were insulin challenged and hepatic expression of (**f**) pAkt (WT: *n* = 4, Scly^−/−^: *n* = 7); (**g**) pAMPK (*n* = 4/group); and (**h**) pACC1 (*n* = 4/group) was measured. Phosphorylated proteins were normalized to total protein levels. All data are represented as means ± S.E.M (* *p* < 0.05, *** *p* < 0.001).

We previously reported that male Scly^−/−^ mice develop a metabolic syndrome-like phenotype that is exacerbated when Se supply is limited [1]. Female Scly^−/−^ mice exhibited a milder phenotype, and thus were not extensively characterized in the previous study after initial cursory examination. In the present study, female Scly^−/−^ and WT mice were fed a low Se diet from weaning to 20 weeks of age, and analyzed for effects of Se deficiency on metabolic phenotype. At 8 weeks of age, the body weights of female Scly^−/−^ mice were not distinct from their WT counter parts. A time course assessment of body weight revealed a main effect of genotype and time. *Post hoc* analysis showed that by 12 weeks of age, female Scly^−/−^ mice were significantly heavier than their WT counterparts, a trend that persisted up to at least 20 weeks of age (Figure 1a). This time point was chosen because symptoms of metabolic syndrome in male Scly^−/−^ mice begin to appear by 20 weeks of age. Female Scly^−/−^ mice also had a significantly higher percentage of gonadal fat deposition (Figure 1b). We administered an intravenous glucose tolerance test, but found no differences in glucose clearance (Figure 1c,d), further confirming that female Scly^−/−^ mice are insulin sensitive. In contrast to what was previously observed in male Scly^−/−^ mice [1], fasting insulin levels were similar in female Scly^−/−^ and WT mice (Figure 1e). Next, we subjected 22-week-old mice to a 4-hour fast, followed by insulin challenge. We found no changes in phosphorylated Akt at serine 473 (pAkt Ser473), a major mediator of insulin signaling (Figure 1f). No differences were observed in phosphorylated AMP-activated protein kinase alpha at threonine 172 (pAMPKα Thr172; Figure 1g) or phosphorylated acetyl-CoA carboxylase (pACC1; Figure 1h), major regulators of lipid and cholesterol metabolism. Taken together, our results demonstrate that, similar to their male counterparts, maintenance of female Scly^−/−^ mice on a low Se diet results in increased adiposity, but unlike males, female Scly^−/−^ mice do not develop hyperinsulinemia, reduced glucose tolerance, downregulated insulin signaling, or activated de novo lipogenesis.

### 3.2. Castration Does Not Rescue the Metabolic Phenotype of Male Scly^−/−^ Mice

**Figure 2:**
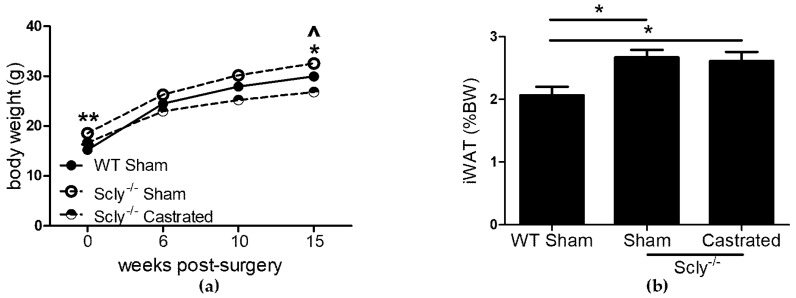
The effects of castration on body and fat weight. Unless indicated, metabolic parameters were measured in mice that were 20–22 weeks of age. For WT sham-operated mice: *n* = 6, Scly^−/−^ sham-operated mice: *n* = 8, Scly^−/−^ castrated mice: *n* = 6. (**a**) Body weight at the indicated time points after surgery. One-way ANOVA was used to test each time point (6 weeks: *F*_2,17_ = 5.88, *p* = 0.01; 10 weeks: *F*_2,17_ = 14.0, *p* = 0.0003; 15 weeks: *F*_2,17_ = 18.66, *p* < 0.0001) and Dunnett’s post-test was used to compare all groups to WT (WT vs. sham: ^ *p* < 0.05, WT vs. castrated: * *p* < 0.05, ** *p* < 0.01); (**b**) inguinal WAT (iWAT) depots displayed as % of bodyweight (one-way ANOVA: *F*_2,17_ = 5.94, *p* = 0.01). All data are represented as means ± S.E.M.

To determine whether Se demands of the male reproductive system contribute to the adverse effects on energy metabolism in male Scly^−/−^ mice, we investigated the effects of castration in a long-term study. Scly^−/−^ mice were castrated at 5-weeks-old and were monitored for 4 months. This time point was chosen because Scly^−/−^ mice on a Se adequate diet are significantly obese at this age [1]. If castration were to rescue energy metabolism dysfunction in male Scly^−/−^ mice, a possible explanation would be that removal of the testes resulted in decreased competition for Se. Six weeks post-surgery, Scly^−/−^ sham-operated mice were heavier than WT sham-operated mice, whereas Scly^−/−^ castrated mice weighed less than WT sham-operated mice. This trend continued at 10 weeks, persisting until at least 15 weeks after surgery (Figure 2a). We predicted that if competition from testes played an adverse effect, body weights of castrated mice would be restored to WT levels. Bonferroni’s post-test following ANOVA revealed that castrated Scly^−/−^ mice indeed weighed less than their sham-operated counterparts at 10 weeks (*p* < 0.001) and 15 weeks (*p* < 0.001) post-surgery. However, castrated mice weighed significantly less than WT mice at both 10 weeks (*p* < 0.05) and 15 weeks (*p* < 0.05) post-surgery. Despite the differences in body weight, both sham-operated and castrated Scly^−/−^ mice had significantly higher inguinal WAT composition when compared to WT sham-operated mice, using Dunnett’s multiple comparison (Figure 2b).

**Figure 3:**
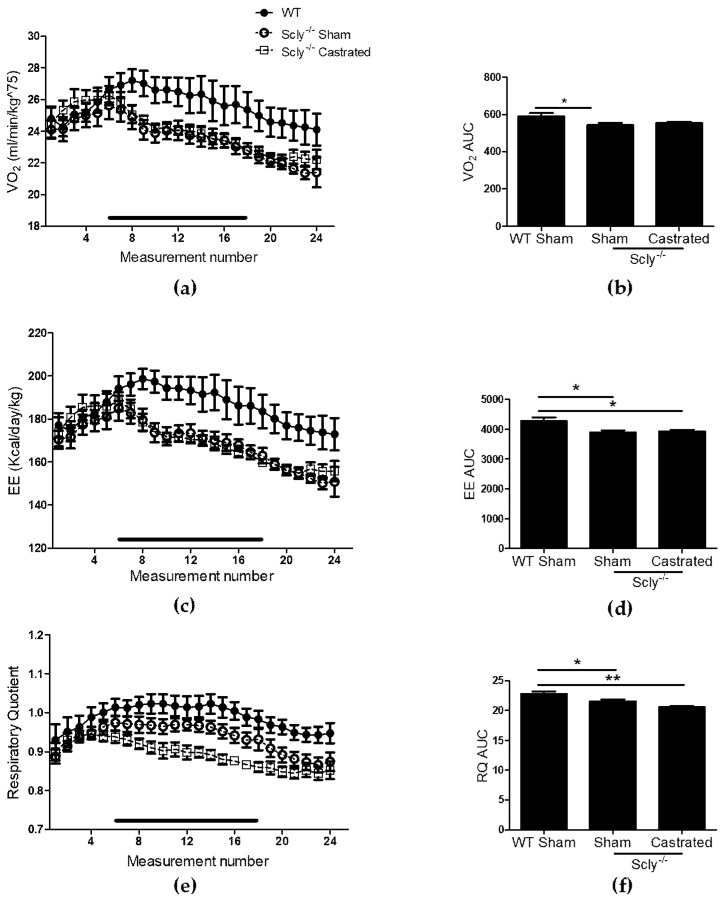
The effects of castration on energy expenditure in 22-week-old mice. Measurements were taken over 24 h, once every hour. Each point represents an average of *n* = 6 for WT and castrated mice, and *n* = 8 for sham-operated mice. The black bar corresponds to measurements taken during the dark cycle. (**a**) VO_2_ profile over 24 h; (**b**) Area under the curve (one-way ANOVA: *F*_2,14_ = 3.48, *p* = 0.06); (**c**) energy expenditure (EE); (**d**) area under the curve for energy expenditure (one-way ANOVA: *F*_2,14_ = 5.76, *p* = 0.01); (**e**) respiratory quotient (RQ) over 24 h; (**f**) area under the curve (one-way ANOVA: *F*_2,14_ = 8.81, *p* = 0.003). Dunnett’s post-test was used to compare all columns to WT (* *p* < 0.05, ** *p* < 0.01). All data are represented as means ± S.E.M.

Indirect calorimetry revealed significant differences in VO_2_ when comparing WT to Scly^−/−^ sham-operated mice and a similar trend when comparing WT to castrated mice, although values did not reach significance (Figure 3a,b). Both groups of Scly^−/−^ mice had decreased energy expenditure (Figure 3c,d) and respiratory quotient (RQ; Figure 3e,f) when compared to WT.

### 3.3. Castration Restores Fasting Serum Insulin, Which Is Not Due to Changes in Islet Size

**Figure 4:**
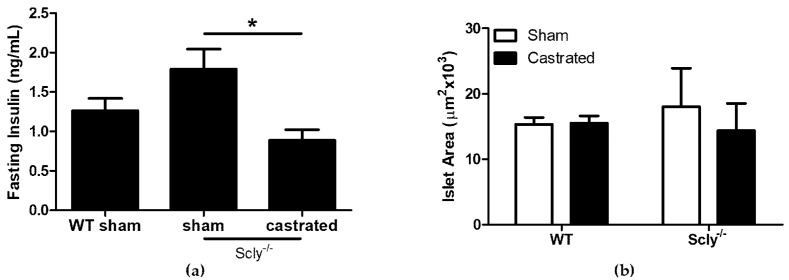
Castration effects on fasting serum insulin. All parameters were measured using 5-month-old mice. (**a**) Serum insulin levels after a 4 h fast (one-way ANOVA: *F*_2,17_ = 5.14, *p* = 0.01). For WT sham mice: *n* = 6, Scly^−/−^ sham mice: *n* = 8, Scly^−/−^ castrated mice: *n* = 6 (* *p* < 0.05). (**b**) Average islet area (two-way ANOVA: interaction *F*_1,11_ = 0.19, *p* = 0.67; genotype *F*_1,11_ = 0.03, *p* = 0.86; castration *F*_1,11_ = 0.15, *p* = 0.70. For WT mice: *n* = 3, Scly^−/−^ sham mice: *n* = 4, Scly^−/−^ castrated mice: *n* = 6. All data are represented as means ± S.E.M.

Remarkably, Scly^−/−^ castrated mice exhibited lower fasting insulin levels when compared to Scly^−/−^ sham mice (Figure 4a). We measured islet area in sham-operated and castrated WT and Scly^−/−^ mice, but found no differences between the groups (Figure 4b). Thus, the reduced fasting serum insulin observed in Scly^−/−^ castrated mice cannot be explained by differences in islet area.

### 3.4. Hypothalamic Selenoprotein Expression Decreases in Both Male and Female Scly^−/−^ Mice

**Figure 5:**
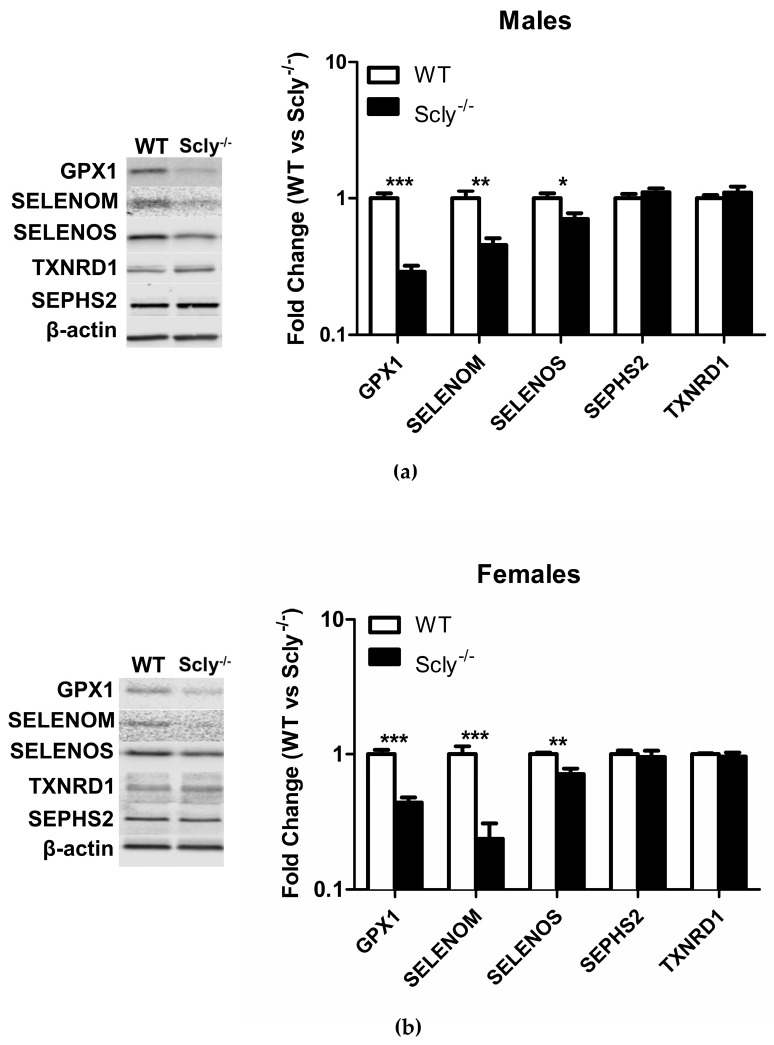
Hypothalamic selenoprotein expression in WT vs. Scly^−/−^ mice. (**a**) Hypothalamic selenoprotein expression in male WT vs. Scly^−/−^ mice, *n* = 6 and (**b**) female WT vs. Scly^−/−^ mice, *n* = 5. β-actin expression was used as a loading control. WT protein expression was normalized to 1. Unpaired, two-tailed *t*-test was used to compare WT vs. Scly^−/−^ for each protein (* *p* < 0.05, ** *p* < 0.01, *** *p* < 0.001). All data are represented as means ± S.E.M.

Recently, it was reported that loss of selenoproteins in the hypothalamus through a conditional knockout of selenocysteine-specific tRNA (Sec-tRNA^(Ser)Sec^) resulted in T2D in mice [10]. Thus, we measured selenoprotein levels in the hypothalamus of male and female Scly^−/−^ mice under Se adequate conditions, using Western Blotting. In males, Scly^−/−^ GPX1 (glutathione peroxidase 1) levels were reduced to 29% ± 0.03 of the levels found in WT hypothalamus, SELENOM (selenoprotein M) was reduced to 46% ± 0.05, and SELENOS (selenoprotein S) was reduced to 71% ± 0.18 (Figure 5a). In female mice, GPX1 expression was at 44% ± 0.04, SELENOM levels at 24% ± 0.07, and SELENOS at 72% ± 0.07 of WT hypothalamus levels. Interestingly, male Scly^−/−^ GPX1 levels were reduced to a greater extent than females (29% vs. 44% of WT), whereas the inverse was true for SELENOM (males, 46% vs. females, 24% of WT). We also measured expression of housekeeping selenoproteins SEPHS2 (selenophosphate synthetase 2) and TXNRD1 (thioredoxin reductase 1), which are more resistant to changes in Se status [7]. As expected, expression of SEPHS2 and TXNRD1 were unchanged (Figure 5b).

## 4. Discussion

Sex differences in incidence of T2D in humans following Se supplementation have been previously noted in a number of studies in which increased Se intake correlated with T2D in men but not women [7]. However, the mechanism behind the sex differences in the relationship between Se and energy metabolism is unclear. We previously observed that female Scly^−/−^ mice do not develop metabolic syndrome with the same frequency or to the same extent as their male counterparts. Thus, we wondered whether the sexually dimorphic outcome of Se in T2D could be attributed to sex-specific regulation of Scly. In order to answer this question, we first characterized female Scly^−/−^ mice. Our results revealed that in accordance with our previous observations, unlike their male counterparts, female Scly^−/−^ mice have normal glucose tolerance, fasting insulin levels, and appear to have normal hepatic insulin signaling and de novo lipogenesis (Figure 1c–h) despite higher body weight and adiposity, when compared to WT mice (Figure 1a,b). The protective effects of estrogen against insulin resistance have been well documented [13]. For instance, pre-menopausal women are protected from the impacts of a high fat diet on insulin resistance when compared to age-matched men, but these effects are abolished in menopausal women [14]. Additionally, estrogen replacement has been shown to directly improve insulin sensitivity [15]. Thus, we suspect that the presence of estrogen may have masked the effects of increased adiposity on insulin resistance in female Scly^−/−^ mice.

Scly was previously found to be expressed in spermatocytes and the Leydig cells of the testes [4]. In addition, castration of Scly^−/−^SELENOP^−/−^ mice attenuated their severe neurological phenotype, suggesting Se distribution to the testes is prioritized over distribution to brain and other tissues [6]. Together, these results reinforce the importance of Se delivery and recycling to testes function and male fertility. Thus, the possibility that the Se demands of the testes produce adverse effects on the function of metabolic tissues in Scly^−/−^ mice could not be excluded. We therefore castrated Scly^−/−^ mice to evaluate whether testes removal could rescue the metabolic dysfunction in Scly^−/−^ mice. Without the testes, circulating Se would be more readily available for distribution to metabolic tissues.

Body fat composition was higher in Scly^−/−^ mice than in WT mice (Figure 2a), and indistinguishable between sham-operated and castrated Scly^−/−^ mice, despite the lower body weight of the castrated mice (Figure 2b). Strikingly, we found castration to significantly reduce fasting serum insulin levels (Figure 4a). This result may be the outcome of either of two possible explanations. On one hand, it is possible that castration rescued insulin sensitivity and β-cell function, eliminating the need for hyperinsulinemia as a compensatory mechanism, thus resulting in a rescued metabolic phenotype. If this were the case, we would likely see improved body fat composition and energy expenditure in castrated Scly^−/−^ mice; however, none of these were observed. Thus, it is more likely that altered insulin secretion in castrated Scly^−/−^ mice is due to β-cell decompensation, which is characterized by a reduction of β-cell mass [16]. Perplexingly, we did not observe differences in islet area in sham-operated and castrated Scly^−/−^ mice (Figure 4b). Further investigation is necessary to determine the effects of castration on fasting serum insulin levels when Scly is absent.

Our finding that body fat composition is higher in Scly^−/−^ than WT mice, regardless of sex (Figure 1b and Figure 2b), points to a likely involvement of Scly in lipogenesis and/or lipolysis. However, we previously reported Scly was expressed minimally in the WAT when compared to the liver. Another study which measured Scly activity in rat tissues failed to detect Scly activity in the WAT [1,8], indicating that the regulatory action of fat stores through Scly is likely indirect. We demonstrate that even on a Se adequate diet, expression of selenoproteins was diminished in the hypothalamus when Scly^−/−^ is absent (Figure 5). This is in contrast to a previous study from our laboratory which found selenoprotein mRNA from whole brain to be unchanged in Scly^−/−^ mice [11], suggesting that Scly may play a more prominent role in selenoprotein biosynthesis in certain brain regions than others.

Crosstalk between the hypothalamus and WAT is well documented, and mostly attributed to the adipokine leptin [17]. Recently, it was found that direct sympathetic efferent contact with the WAT is critical to the action of leptin on lipolysis via the hypothalamus [18]. Thus, it is tempting to speculate that Scly influences WAT function by regulating selenoprotein expression in the hypothalamus. In support of this, a recent study found that deletion of Sec-tRNA^(Ser)Sec^ from the hypothalamus results in increased adiposity and insulin resistance [10]. The effects were found to be a direct result of increased oxidative stress due to the absence of Sec-tRNA^(Ser)Sec^.

We previously showed that SELENOM knockout mice develop obesity and decreased leptin signaling [19]. Interestingly, SELENOM is one of the selenoproteins that we found to be downregulated in the hypothalamus of Scly^−/−^ mice. The reduction in hypothalamic selenoprotein expression was observed at 10-weeks-old of age, prior to significant changes in energy metabolism. Thus, diminished selenoprotein expression is likely a contributing factor to alterations in energy metabolism, rather than a consequence. This invites speculation that Scly may protect against alterations in lipid metabolism, through the regulation of select selenoproteins. One additional selenoprotein that may be regulated by Scly is type 2 iodothyronine deiodinase (DIO2) [20]. The iodothyronine deiodinases are selenoenzymes with the purpose of regulating thyroid hormone activation. Whole body deletion of DIO2 in mice results in susceptibility to diet-induced obesity [21]. Potentially, Scly indirectly regulates energy metabolism in a DIO2-dependent manner, an effect that would have impact on metabolic pathways that are thyroid hormone-dependent. Moreover, we found an apparent sexual dimorphism in the extent of reduction in hypothalamic GPX1 and SELENOM in Scly^−/−^ mice. Thus, it is possible that these differences in hypothalamic selenoprotein regulation can contribute to sex differences in Scly^−/−^ mice.

Taken together, our results suggest a novel role for Scly in regulating energy metabolism through the hypothalamus. Scly appears to be critical for efficient hypothalamic selenoprotein biosynthesis, particularly for the Se-responsive selenoproteins. An interesting avenue for further investigation would be to determine whether hypothalamic Scly protects against high fat diet and whole-body insulin resistance. Moreover, our castration studies demonstrate that Se sequestration by the testes does not account for the sexual dimorphism observed in Scly^−/−^ mice.
